# The causal relationship between diet habits and cholelithiasis: a comprehensive Mendelian randomization (MR) study

**DOI:** 10.3389/fnut.2024.1377631

**Published:** 2024-06-12

**Authors:** Lin Xie, Mingzhi Xu, Yahan Lei, Juan Li, Jiajia Xie

**Affiliations:** ^1^The Seventh Clinical Medical College of Guangzhou University of Chinese Medicine, Shenzhen, Guangdong, China; ^2^Shenzhen Bao’an Chinese Medicine Hospital, Guangzhou University of Chinese Medicine, Shenzhen, Guangdong, China

**Keywords:** diet, dried fruit intake, cholelithiasis, Mendelian randomization, sensitivity

## Abstract

**Background:**

Epidemiological studies show dietary habits can have an impact on the risk of cholelithiasis, but the relationship is still unclear. We used a comprehensive Mendelian randomization (MR) study to explore the relationship between dietary habits and cholelithiasis.

**Methods:**

The 18 dietary habits were divided into six categories: meat foods, cereals, vegetables, fruits, dairy products, beverages, and condiments. Cholelithiasis data came from a GWAS meta-analysis and the FinnGen consortium. The inverse variance weighted (IVW), the weighted median (WM), and MR-Egger approaches were used as the main MR analysis methods. In addition, multiple sensitivity analysis and meta-analysis were performed to verify the robustness of the results.

**Results:**

Dried fruit intake [odds ratio (OR) = 0.568; 95% confidence interval (CI), 0.405–0.797; *p* = 0.001] was discovered to reduce the risk of cholelithiasis. The sensitivity analysis and meta-analysis showed reliable results for the relationship between dried fruit intake and cholelithiasis.

**Conclusion:**

Our study found that dried fruit intake is a protective factor in the development of cholelithiasis. However, the mechanisms of action need to be further explored.

## Introduction

Cholelithiasis is a common gastrointestinal disorder that usually has no clinical symptoms (1). Cholelithiasis affects up to 20% of the population in Europe and can cause a loss of up to $1.6 billion per year (2, 3). Studies have shown that 20% to 35% of asymptomatic patients will develop symptomatic cholelithiasis during their lifetime and more than 30,000 people are hospitalized for cholelithiasis each year (3–5). Cholecystectomy is the primary treatment for cholelithiasis, with over 830,000 cholecystectomies carried out annually in the United Kingdom and the United States (6, 7). However, many patients with cholelithiasis do not benefit from cholecystectomy, and the complications of this treatment may reduce the patient’s overall quality of life (6, 8, 9). Gastrointestinal dysfunction and chronic pain are common postoperative complications (10–13). In terms of medication, using generic medications to prevent gallstones is not recommended, even if predisposing factors are present (2). Ursodeoxycholic acid, a commonly used drug for the treatment of cholelithiasis, should only be used in patients with occasional small stones with symptoms (2). Meanwhile, there is controversy in different studies regarding ursodeoxycholic acid for cholelithiasis (14, 15). Therefore, it is necessary to prevent cholelithiasis through modifiable factors (2).

Nutrition intervention, as an important means to intervene in stone development, has the potential to reduce the occurrence of cholelithiasis and promote therapeutic intervention (16, 17). Many recent studies have indicated that dietary factors are linked to cholelithiasis (18, 19). Consuming carbohydrates and saturated fats may increase the risk of forming gallstones. Consumption of protein, fiber, nuts, coffee, and moderate amounts of alcohol may reduce this (20). Moreover, an animal study suggests that a phosphatidylcholine diet helps prevent the formation of gallstones (21). However, the results of observational studies may not be completely reliable because of reverse causality and confounding factors (22). Therefore, it is still necessary to explore the relationship between dietary habits and cholelithiasis. To correctly and reliably assess the relationship between diet and cholelithiasis, MR methods were performed.

MR is a method of epidemiologic investigation that relies on genetic variation to distinguish between observed correlation and causality (23). Meanwhile, MR analysis can generate robust evidence for which interventions should yield health benefits through modifiable exposure to closely related genetic variations (24). It overcomes the shortcomings of randomized controlled trials that are costly, time-consuming, and less feasible (25).

While MR designs have been used to explore the relationship between dietary factors and the risk of a diverse range of diseases, MR analyses of the relationship between dietary factors and cholelithiasis have not yet been performed (26, 27). A comprehensive exploration of the role of dietary habits in cholelithiasis is crucial for the development of nonpharmacologic interventions. This study used a comprehensive MR approach to assess the effects of dietary habits on cholelithiasis.

### Study design

**FIGURE 1:**
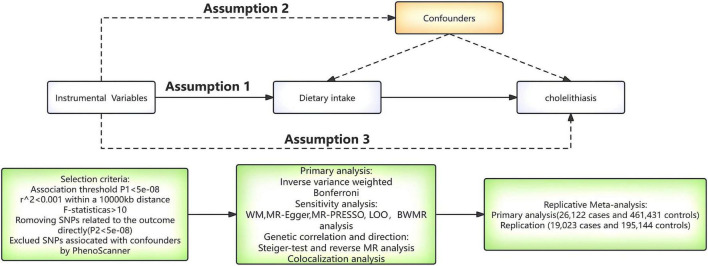
A flow chart of the study. WM, weighted median; MR-PRESSO, MR polytropic residual sums and outliers; LOO, leave-one-out; BWMR, Bayesian weighted Mendelian randomization.

Figure 1 provides a flow chart of our study. A comprehensive MR approach was performed to explore the potential effects of dietary habits on cholelithiasis. The MR analysis should meet the three core hypotheses: (1) genetic variable tools are strongly correlated with dietary habits (28); (2) genetic variable tools should be independent of any confounding factors related to cholelithiasis (29); (3) genetic variable tools can only influence cholelithiasis through dietary habits (30). Notably, the sample size had an impact on the estimates of the MR analysis, we used two GWAS data, one for primary analysis and the other for repeated analyses to increase the confidence of the results.

### Genome-wide association study (GWAS) data for dietary habits and cholelithiasis

We collected the GWAS data for dietary habits and cholelithiasis from the IEU Open GWAS Project.^1^

The GWAS data for 18 dietary habits were derived from the UK Biobank, a large population-based survey of genetic and non-genetic factors for disease in middle-aged and older adults (31). The 18 dietary habits were divided into six categories: meat foods (processed meat, beef, mutton, pork, non-oily fish, oily fish, poultry, Lamb/mutton); cereals (cereals, bread); vegetables (salad/raw vegetables, cooked vegetables); fruits (dried fruit, fresh fruit); dairy products (cheese); beverages (coffee, tea, alcohol), and condiments (salt). The GWAS data for cholelithiasis were obtained from two datasets: (1) the pooled data for cholelithiasis for the main analysis came from a GWAS meta-analysis of a mixed population including 26,122 cases and 461,431 controls (32). (2) The second cholelithiasis GWAS data were derived from the FinnGen consortium,^2^ including the number of 19,023 cases and 195,144 controls (Table 1).

**TABLE 1:** The information of GWAS datasets on dietary habits and cholelithiasis.

Trait	Sample size	Consortium	GWAS ID
Processed meat intake	461,981	MRC-IEU	ukb-b-6324
Poultry intake	461,900	MRC-IEU	ukb-b-8006
Beef intake	461,053	MRC-IEU	ukb-b-2862
Non-oily fish intake	460,880	MRC-IEU	ukb-b-17627
Oily fish intake	460,443	MRC-IEU	ukb-b-2209
Pork intake	460,162	MRC-IEU	ukb-b-5640
Lamb/mutton intake	460,006	MRC-IEU	ukb-b-14179
Bread intake	452,236	MRC-IEU	ukb-b-11348
Cereal intake	441,640	MRC-IEU	ukb-b-15926
Cheese intake	451,486	MRC-IEU	ukb-b-1489
Alcohol intake frequency	462,346	MRC-IEU	ukb-b-5779
Tea intake	447,485	MRC-IEU	ukb-b-6066
Coffee intake	428,860	MRC-IEU	ukb-b-5237
Cooked vegetable intake	448,651	MRC-IEU	ukb-b-8089
Salad / raw vegetable intake	435,435	MRC-IEU	ukb-b-1996
Fresh fruit intake	446,462	MRC-IEU	ukb-b-3881
Dried fruit intake	421,764	MRC-IEU	ukb-b-16576
Salt added to food	462,630	MRC-IEU	ukb-b-8121
Cholelithiasis	487,553		GCST90018819
Cholelithiasis	214,167	the FinnGen consortium	finn-b-K11_CHOLELITH

### Selection of instrumental variables (IVs)

We employed the following criteria to select the single nucleotide polymorphisms (SNPs) as the valid instrumental variables: (1) we selected SNPs associated with dietary habits (*p* < 5e-08), making sure they are independent of an aggregate distance of 10,000 kb (*r*^2^ < 0.001); (2) The SNPs strongly associated with cholelithiasis (*p* < 5e-08) were deleted; (3) We tested for associations between instrumental variables and dietary habits using formula F. When F is greater than 10, instrumental variables are considered to effectively avoid bias from weak instruments (33). (4) A palindromic SNP with an intermediate allele frequency was excluded from the analysis to maintain the consistency between the effects of the SNPs on the exposure and the outcome. (5) We removed those SNPs that came out by the MR polytropic residual sums and outliers (MR-PRESSO) test as potentially affecting the results. (6) Since body mass index (BMI) (34), diabetes (35), and cholesterol level (36) were risk factors for the formation of cholelithiasis, we excluded SNPs associated with BMI, diabetes, Triglycerides, and total cholesterol by the PhenoScanner database (Supplementary Table 1).^3^

### Univariate MR analysis

The IVW method is the main method for MR analysis and provides reliable results in the absence of horizontal pleiotropy (37). To improve the reliability of the evaluation results, we used the WM method and the MR-Egger method as a complement to the IVW method (38, 39). The Cochran’s Q test was used to test for heterogeneity, and the MR-Egger intercept was used to assess horizontal pleiotropy (40–42). When heterogeneity or multiplicity was present (*p* < 0.05), We recognized potential outliers using the MR-PRESSO analysis. MR-PRESSO analysis is used to detect and attempt to reduce level pleiotropy by excluding significant outliers (43). After excluding the outliers, MR analysis was performed again. The leave-one-out (LOO) analysis was used to assess the effect of a single SNP on the outcome (40). Due to multiple testing, the Bonferroni correction (0.003, 0.05/18) was used to adjust the *p*-value (44).

### Bayesian weighted Mendelian randomization (BWMR)

For the significant dietary habits, we performed the BWMR analysis for the evaluation. BWMR considers the uncertainty of weak effects due to the polygenic structure of complex traits, and the problem of violating IV assumptions due to polygenicity (45).

### Directionality test and reverse MR analysis

We used the Steiger test and reverse MR analysis to assess whether cholelithiasis also influenced dietary habits. The Steiger test can be used to confirm whether the observed causality deviates due to reverse causality (46). Causal inference was not biased when SNP combinations were found to have no genetic risk for cholelithiasis compared to dietary habits (Steiger *p* < 0.05). The reverse MR analysis further assessed whether cholelithiasis showed a causal effect on dietary habits.

### Multivariate MR analysis (MVMR) and colocalization analysis

Previous MR studies suggest that there may be reciprocal influences because dietary habits are not independent factors (47). We performed a multivariate analysis of the identified dietary factors to assess whether there was a mutual influence between each other. Furthermore, we applied colocalization analysis to test whether the identified dietary habits and cholelithiasis share common causal variants in a given region (48). Based on previous studies, the significant colocalization (posterior probability) was set to PP.H4 > 0.95. When PP.H4 > 0.95, exposure was considered a potential contributing factor (49).

### Meta-analysis

For dietary habits significantly associated with cholelithiasis, we used two different GWAS-related data to assess the robustness of our results.

### Statistical analysis

We used R software (version 4.3.2) to analyze. The TwoSampleMR (version 0.5.7), color (version 5.2.3), meta (version 6.5-0), and MR-PRESSO (version 1.0) packages were included.

## Results

Following a rigorous instrument selection procedure, we performed an MR analysis of 18 dietary habits. All the F- statistics exceed the empirical threshold of 10 (Supplementary Table 2).

### Univariate MR analysis

**FIGURE 2:**
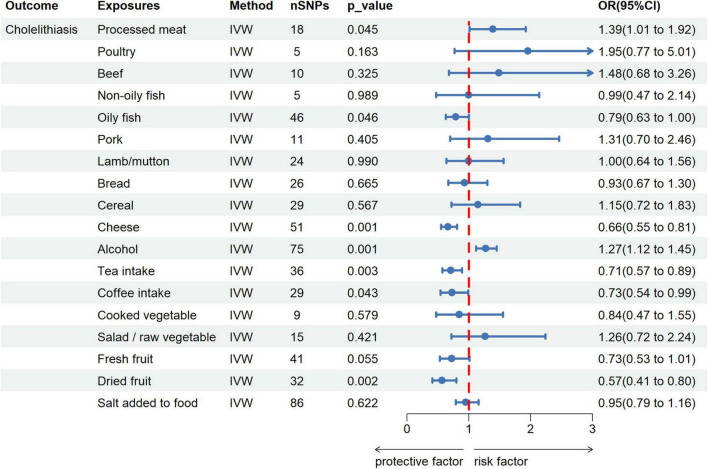
Forest plot for the causal effect of dietary habits on the risk of cholelithiasis. IVW, inverse variance weighted; SNPs, single nucleotide polymorphisms; OR, odds ratio; 95% CI, 95% confidence interval.

After the Bonferroni correction (*p* < 0.003), 4 dietary habits were initially identified by IVW as significantly related to cholelithiasis. Among them, intake of cheese (OR = 0.661; 95% CI, 0.542–0.808; *p* = 5.02 × 10^−5^), tea (OR = 0.707; 95% CI, 0.566–0.886; *p* = 0.002), and dried fruit (OR = 0.568; 95% CI, 0.405–0.797; *p* = 0.001) reduced cholelithiasis scores. In contrast, alcohol intake (OR = 1.272; 95% CI, 1.120–1.446; *p* = 0.0002) increased cholelithiasis scores (Figure 2 and Supplementary Table 3). The direction and amplitude of the WM and MR-Egger methods remained consistent with the IVW method, which supported the robustness of the causal relationships. The results of the scatter plots indicated the stability of the results (Supplementary Figure 1). The *p*-value of 4 dietary habits in the MR-Egger intercept were greater than 0.05, which implied that there was no horizontal pleiotropy (Supplementary Table 4). LOO analysis also did not find any SNP with a strong impact on the outcome (Supplementary Figure 2).

### BWMR

BWMR showed cheese intake (OR = 0.669; 95% CI, 0.545–0.821; *p* < 0.001), alcohol intake (OR = 1.297; 95% CI, 1.145–1.470; *p* < 0.001), tea intake (OR = 0.713; 95% CI, 0.567–0.897; *p* = 0.003) and dried fruit intake (OR = 0.556; 95% CI, 0.398–0.776; *p* < 0.001) were association with cholelithiasis (Table 2).

**TABLE 2:** The results of BWMR analysis.

Exposures	Method	Beta	OR	95% CI	*p*-value
Cheese intake	BWMR	−0.402	0.669	0.545–0.821	0.000116
Alcohol intake	BWMR	0.260	1.297	1.145–1.47	0.0000449
Tea intake	BWMR	−0.338	0.713	0.567–0.897	0.003789
Dried fruit intake	BWMR	−0.587	0.556	0.398–0.776	0.000557

BWMR, Bayesian weighted Mendelian randomization; OR, odds ratio; 95% CI, 95% confidence interval.

### Directionality test and reverse MR analysis

The results of the Steiger test did not support a reverse causal effect between dietary habits and cholelithiasis (*p* < 0.05). Furthermore, reverse MR analysis indicated no association of cholelithiasis with cheese intake (*p* = 0.114), tea intake (*p* = 0.117), dried fruit intake (*p* = 0.424), and alcohol intake (*p* = 0.674) (Supplementary Table 5).

### MVMR and colocalization analysis

**FIGURE 3:**
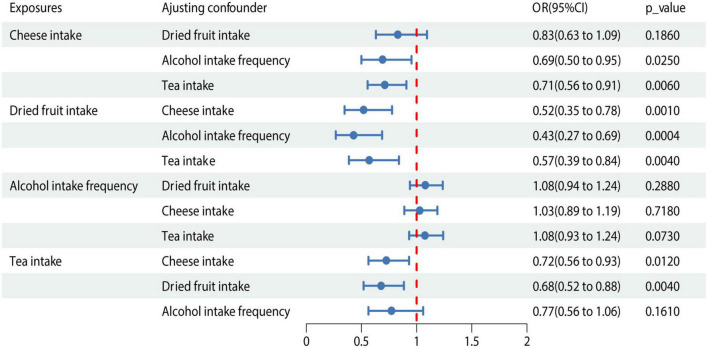
MVMR analysis of the 4 dietary habits. OR, odds ratio; 95% CI, 95% confidence interval.

We conducted MVMR analysis of the 4 dietary habits according to the causality determined by the IVW method described above (Figure 3). The association between dried fruit intake and cholelithiasis was still significant in MVMR analysis when adjusted for cheese intake (OR = 0.519; 95% CI, 0.347–0.776; *P* = 0.001), alcohol intake (OR = 0.428; 95% CI, 0.267–0.687; *P* < 0.001), and tea intake (OR = 0.569; 95% CI, 0.386–0.839; *P* = 0.004). Colocalization analysis revealed that dried fruit intake and cholelithiasis shared a causal variant (PP.H4 = 0.952) within the gene region (± 500 kb). Meanwhile, no causal variant shared cheese intake (PP.H4 = 8.74 × 10^−19^), tea intake (PP.H4 = 0.002), and alcohol intake (PP.H4 = 0.007) with cholelithiasis (Supplementary Table 6).

### Meta-analysis

**FIGURE 4:**
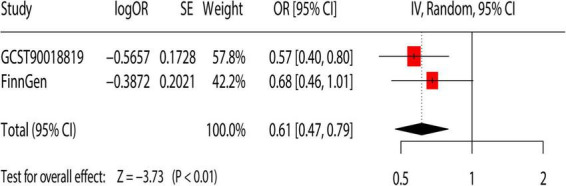
Meta-analysis of the causal association between dietary habits and cholelithiasis. OR, odds ratio; 95% CI, 95% confidence interval; SE, standard error. GCST90018819: Primary analysis of cholelithiasis GWAS; FinnGen: replication analysis of cholelithiasis.

We performed repeated validation using additional GWAS dataset to further confirm the causal relationship between dried fruit intake and cholelithiasis. The result showed that the higher intake of dried fruit intake (OR = 0.61; 95% CI, 0.47–0.79; *p* < 0.01) was associated with a lower risk of cholelithiasis (Figure 4).

## Discussion

We used large-scale GWAS data to assess the impact of 18 dietary habits on the incidence of cholelithiasis. Among the 18 dietary habits, IVW analysis and Bonferroni correction initially identified causal associations between tea intake, cheese intake, dried fruit intake, and alcohol intake and cholelithiasis. MVMR analysis and colocalization analysis indicated that among the 4 dietary habits, dried fruit intake had the most reliable association with cholelithiasis. Finally, the result of the meta-analysis confirmed that a higher intake of dried fruits is associated with a reduced risk of cholelithiasis.

Early cholelithiasis is usually asymptomatic, which increases the difficulty of physician diagnosis and treatment (50). As an independent risk factor for gallbladder cancer, early prevention and intervention should be carried out (51). Dried fruits are healthy snacks for fresh fruit obtained through a variety of drying techniques (52). Dried fruit has a similar nutritional composition to fresh fruit, but it overcomes the defect of the short shelf life of fresh fruit (53). Dried fruit is rich in essential health-promoting substances and nutrients that have an impact on human health (52). Previous studies have linked dried fruit intake to cardiovascular disease, gastrointestinal health, cancer, bone health, etc (54, 55). Our study confirmed from the genetic level that the intake of dried fruit was negatively related to the incidence of cholelithiasis. Multiple sensitivity analyses strongly supported our findings. Therefore, it should be actively advocated that patients with cholelithiasis can appropriately increase their dried fruit intake through dietary intervention to reduce the risk of cholelithiasis.

Studies have indicated that dried fruits are rich in dietary fiber (56). Excretion of bile acids and cholesterol synthesis are crucial steps in the formation of cholelithiasis (57). By promoting the excretion of fecal neutral sterols, dietary fiber can reduce cholesterol (58). Furthermore, supplementation with dietary fibers diminishes the conversion of primary bile acids to secondary bile acids (59). A vitro study found that different types and shapes of raisins have the ability to have bile acids bound to them (60). Our study further confirms the relevance of dried fruit intake in reducing cholelithiasis at the genetic level. However, the potential mechanism of reducing cholelithiasis with dried fruit is currently unclear. More research is needed to further validate the protective mechanisms of dried fruit intake.

Our study is the first MR study to systematically assess the causal relationship between dietary intake and cholelithiasis. We performed strict quality control conditions and used a variety of models to assess causal effects. Furthermore, we used a meta-analysis to validate the credibility of the results. However, there are some shortcomings in our study: (1) All genomic analysis data on dietary factors and cholelithiasis were obtained from the Western populations, and the results would not be extended to other cohorts. (2) We only included 18 dietary factors as exposure, while other dietary factors were not included in the study due to the number of SNPs. (3) Despite our attempts to reduce the bias of confounding factors, some bias may still exist. (4) Due to database limitations, we were only able to determine that dried fruit intake was associated with a reduced risk of cholelithiasis at the genetic level, but we were unable to estimate the ideal amount of dried fruit. (5) The overlap of populations may have some impact on the effect values of the meta-analysis. (6) We found a potential link between dried fruits and cholelithiasis at the gene level. However, studies on dried fruit intake and cholelithiasis are lacking. Therefore, the MR findings should be further verified.

## Conclusion

In conclusion, we found that high levels of dried fruit intake help reduce the incidence of cholelithiasis. Further exploration of conservation mechanisms for the intake of dried fruits is needed.

## Data availability statement

The original contributions presented in this study are included in the article/Supplementary material, further inquiries can be directed to the corresponding author.

## Ethics statement

Due to publicly available GWAS summary statistics, there was no need to apply for ethical approval.

## Author contributions

LX: Writing – original draft. MX: Methodology, Writing – original draft. YL: Methodology, Software, Writing – review and editing. JL: Software, Writing – original draft. JX: Supervision, Writing – review and editing.

## Footnotes

1 https://gwas.mrcieu.ac.uk/, accessed on 12 December 2023

2
https://r5.finngen.fi/

3 http://www.phenoscanner.medschl.cam.ac.uk/, accessed on 12 December 2023
