# Supplementary material for: The causal relationship between diet habits and cholelithiasis: a comprehensive Mendelian randomization (MR) study
Source: Front Nutr. 2024 Jun 12;11:1377631. doi: 10.3389/fnut.2024.1377631 (PMC11203601; doi:10.3389/fnut.2024.1377631)

**The Causal Relationship Between Diet Habits And Cholelithiasis: A Comprehensive Mendelian Randomization (MR) Study**

Author list:

Lin Xie[1], Mingzhi Xu[1] , Yahan Lei[1], Juan Li[1], and Jiajia Xie*[2]

Affiliations:

[1]The seventh clinical medical college of Guangzhou University of Chinese Medicine, Shenzhen, Guangdong Province, 518000 China

[2]Shenzhen Bao’an Chinese Medicine Hospital, Guangzhou University of Chinese Medicine, Shenzhen, Guangdong Province, 518000 China

Corresponding author:

Jiajia Xie,

*Correspondence: xiejiajiabazyy@163.com

Supplementary Figure 1. Scatterplots for the significant association between dietary Habits and cholelithiasis. (A): cheese intake; (B): alcohol intake frequency; (C): tea intake; (D): dried fruit intake.

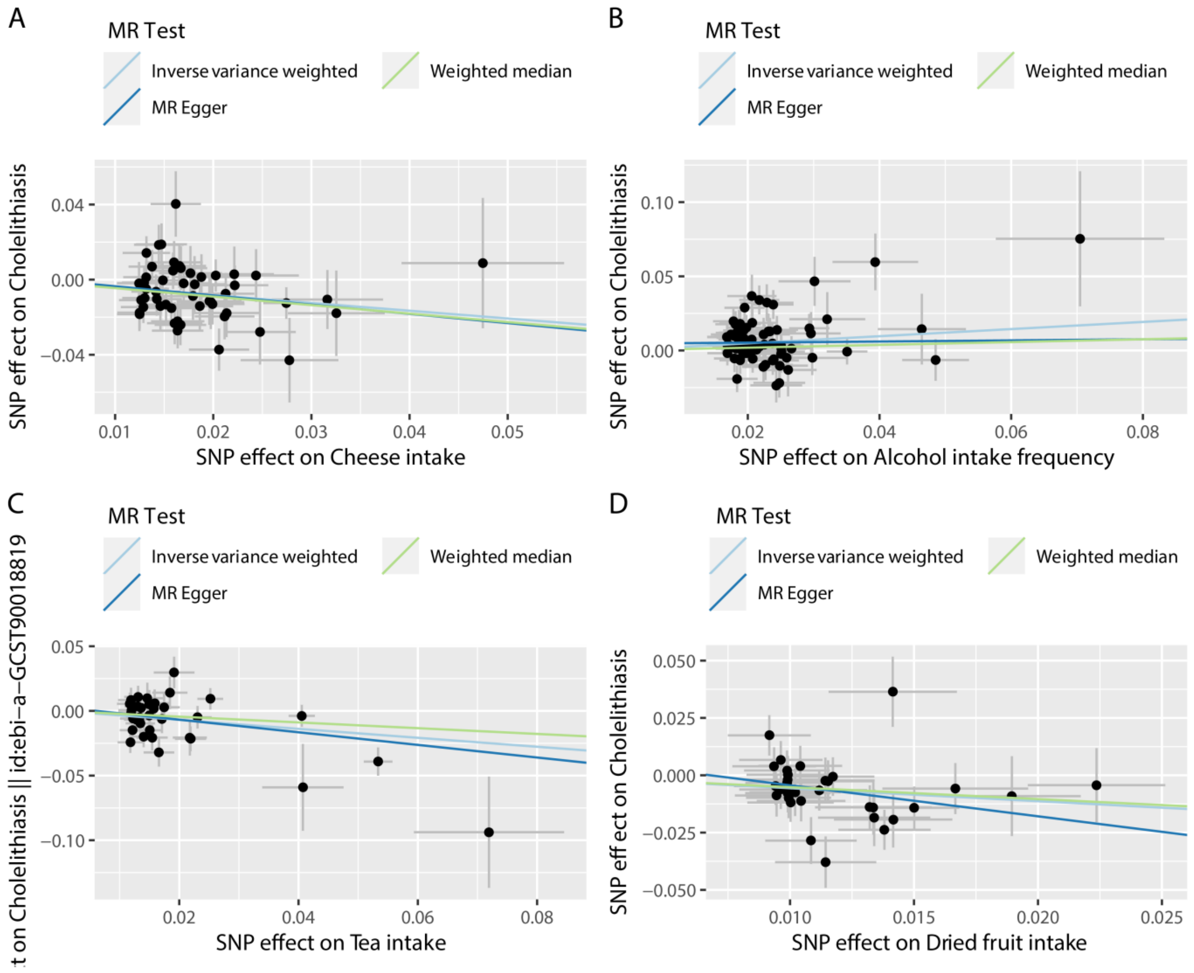

Supplementary Figure 1. The leave-one-out analysis of the significant MR association between dietary habits and cholelithiasis. (A): cheese intake; (B): alcohol intake frequency; (C): tea intake; (D): dried fruit intake.

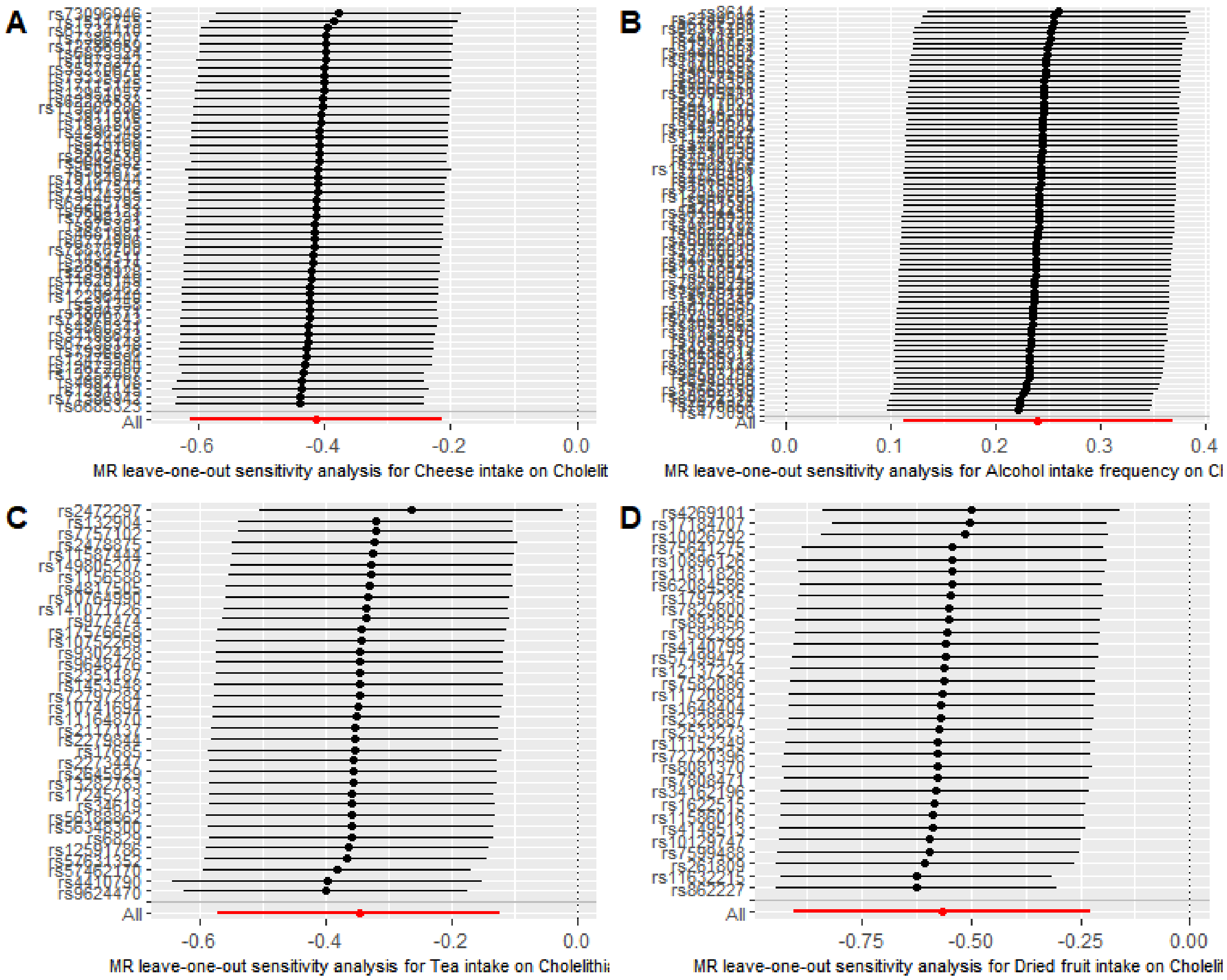

Supplement: Supplementary file 1 [file Image_1.pdf]
